# Clinical and radiological features driving patient selection for antiangiogenic therapy in non-small cell lung cancer (NSCLC)

**DOI:** 10.1186/s40644-016-0102-4

**Published:** 2016-12-28

**Authors:** Cesare Gridelli, Andrea Camerini, Giovanni Pappagallo, Angelo Pennella, Michele Anzidei, Massimo Bellomi, Roberta Buosi, Rosario Francesco Grasso

**Affiliations:** 1 Division of Medical Oncology, S. G. Moscati Hospital, Contrada Amoretta 8, 83100 Avellino, Italy; 2 Medical Oncology, Versilia Hospital and Istituto Toscano Tumori, Lido di Camaiore (LU), Italy; 3 Epidemiology & Clinical Trials Office, General Hospital, Mirano (VE), Italy; 4 Psychologist, Psychotherapist, Lecturer at the School of Specialization in Health Psychology, “Sapienza” University, Rome, Italy; 5 Department of Radiology, “Sapienza” University, Rome, Italy; 6 Division of Radiology, Istituto Europeo di Oncologia, Milan, Italy; 7 Department of Oncology, University of Milan, Milan, Italy; 8 Medical Oncology, East Piedmont University, Maggiore della Carità Hospital, Novara, Italy; 9 Department of Radiology, Campus BioMedico University, Rome, Italy

**Keywords:** Non-small cell lung cancer, Antiangiogenic therapy, Pulmonary haemorrhage, Nominal group technique, Delphi questionnaire, Radiological features, Clinical features

## Abstract

**Background:**

The use of antiangiogenic therapy in non-small cell lung cancer (NSCLC) requires thorough evaluation of patient characteristics in order to avoid potential safety issues, particularly pulmonary haemorrhage (PH). The aim of this consensus by a panel of experts was to identify important criteria for the selection of patients with NSCLC who would benefit from antiangiogenic therapy.

**Methods:**

Radiologists and oncologists were selected for the expert panel. The nominal group technique (NGT) and the Delphi questionnaire were used for consensus generation. The NGT consisted of four steps, the result of which was used to set the Delphi questionnaire. A final report was generated based on the opinions of the experts from the panel.

**Results:**

An extremely important prerequisite for the evaluation of an antiangiogenic therapeutic approach in patients with NSCLC was thorough clinical and radiological analysis of the relationships between tumour and vascular or anatomical structures (performed in close co-operation by oncologists and radiologists). The panel identified major parameters to be considered before the use of antiangiogenic treatment, collectively agreeing on the relevance of tumour cavitation, vascular infiltration, endobronchial growth and thromboembolism for chest tumour sites, and of the presence of aneurysms, extra-thoracic bleeding, brain metastases or thrombi for extra-thoracic sites. Moreover, a structured report containing information not only on the tumour but also on the general vascular status is essential to guide the treatment choice The experts agreed that tumour localization in the absence of vessel infiltration, cavitation, and the use of antiplatelet therapy are relevant parameters to be assessed, but their presence should not necessarily exclude a patient from receiving antiangiogenic therapy.

**Conclusion:**

Close co-operation between oncologists and radiologists in the diagnosis, treatment selection, and assessment of response is essential for ensuring therapeutic appropriateness in the NSCLC setting. It should be noted that neither the use of antiplatelet therapy nor tumour localisation are to be considered as contraindications to antiangiogenic treatment.

## Background

Tumour angiogenesis is a hallmark of cancer pathogenesis and operates through several mechanisms, typically mediated by pro-angiogenic factors [1, 2]. Vascular endothelial growth factor (VEGF) is considered to be the most important angiogenic mediator of endothelial cell proliferation and survival [3].

Non-small cell lung cancer (NSCLC) is one of the two major types of lung cancer, accounting for up to 85% of lung cancers and is associated with a 5-year survival rate of 15.9% [4]. Favourable survival outcomes (6-months progression-free survival [PFS] rate: 74%; 95% CI: 57–97) in NSCLC patients have been reported using anti-VEGF antibodies in combination with first-line chemotherapy [5]. Phase III studies have demonstrated the efficacy of the combination treatment with bevacizumab and carboplatin plus paclitaxel in NSCLC: the survival of the group assigned to bevacizumab plus chemotherapy was significantly improved compared with the group assigned to chemotherapy alone, both in a randomized trial by the Eastern Cooperative Oncology Group (ECOG) (median survival: 12.3 months versus 10.3 months, respectively; *p* = 0.003) [6] and in the BEYOND trial (PFS: 9.2 versus 6.5 months, respectively; p˂0.001) [7]. In addition, the AVAiL (Avastin in Lung) phase III study showed that cisplatin/gemcitabine plus bevacizumab (7.5 mg/kg or 15 mg/kg) offers clinical benefit as compared with cisplatin/gemcitabine plus placebo (median PFS: 6.7 versus 6.1 months, respectively, in the low-dose group, *p* = 0.003; 6.5 versus 6.1 months, respectively, in the high-dose group, *p* = 0.03) and is well tolerated in patients with advanced NSCLC [8, 9]. Moreover, in the real world studies SAiL (Safety of Avastin in Lung) [10] and ARIES (Avastin Regimens: Investigation of Treatment Effects and Safety) [11] the safety profile of the combination treatment of bevacizumab plus standard chemotherapy was consistent, with a low incidence of grade ≥3 adverse events of special interest, and comparable with the findings of previous randomized trials. Bevacizumab is the only antiangiogenic agent currently approved for first-line NSCLC treatment and its use in combination with chemotherapy is recommended by international guidelines [12–14].

Severe pulmonary haemorrhage (PH) is a relatively uncommon but potentially fatal adverse event that occurs preferentially in squamous NSCLC; the incidence of grade ≥3 PH reported during antiangiogenic therapy has been 0.7–1.9% in the phase III and real-world cohort studies, where patients with predominantly squamous cell tumours had been excluded [6, 9–11, 15]. Since a phase II study on advanced NSCLC [16] reported for the first time an increased incidence of PH in those with squamous histology, a consensus report by an interdisciplinary panel has suggested that patients with a history of PH or showing squamous NSCLC should not be given antiangiogenic therapy; major blood vessel and bronchial vessel infiltration may be risk factors for PH and there is no clear connection between tumour location or cavitation and PH [15]. The report recommended an individual risk-benefit analysis in NSCLC patients being considered for therapy.

Imaging is essential for diagnosis and staging of NSCLC as it helps in determining the size, location and tumour baseline features [17]. Both Multi-Detector Computed Tomography (MDCT) [18] and the Crabb diameter method, which was shown to improve the determination of the incidence of tumour cavitation compared with the canonical RECIST criteria [19], have proved to be useful tools to provide a more accurate evaluation of tumour characteristics. However, studies have reported great inter-observer inconsistency during the selection of antiangiogenic therapy in patients with NSCLC; a strong collaboration between oncologists and radiologists, based on international standard guidelines, should therefore be encouraged to help facilitate the decision regarding the use antiangiogenic agents in eligible patients [20].

Most importantly, no imaging criteria that can be used for predicting antiangiogenic therapy-associated PH could be clearly identified from the literature. Newer techniques like computed tomography (CT) perfusion imaging, magnetic resonance (MR) perfusion/diffusion imaging or positron emission tomography-computed tomography (PET-CT) do not provide reproducible results across different study centres and are not commonly used in clinical practice. Determining the relevant parameters to be assessed, therefore, is an increasingly important goal for optimal use of antiangiogenic therapy in patients with NSCLC, particularly in light of the development of newer antiangiogenic agents [21]. The aim of this consensus was to identify the criteria that are important for the selection of patients with NSCLC who would benefit from antiangiogenic therapy, by a panel of oncologists and radiologists, using the Nominal Group Technique (NGT) and Delphi consensus methods.

## Methods

### Selection of experts

In order to evaluate potential risk factors of PH and assess the optimal selection criteria for antiangiogenic therapy in patients with NSCLC, the conflicting scientific and clinical evidence was discussed by a group of six Italian specialists (three oncologists and three radiologists). Members of the expert panel were identified under the following series of criteria [22, 23]: a) interest in the topic; b) a high level of knowledge and clinical experience on the topic; and c) motivation to share their knowledge and experience through the Delphi method.

### Consensus methods

The NGT and the Delphi method were used for this study [24], under the guidance of expert methodologists for each technique. The NGT is a method of consensus generation involving a relatively small panel of experts that express their opinions, in a non-interactive way, about a “core question”. After all participants have given their views, a ranking of the opinions is established by voting. Among its advantages, this technique can be administered to non-homogeneous groups (for example, groups composed of specialists from different areas), and is especially suitable to cases where achieving a consensus appears particularly difficult. In the Delphi method, a series of statements to be evaluated is first defined by the experts. A questionnaire based on the statements is then administered, in two or more rounds, to the participants. After each round the methodologist provides a summary of the answers, in an anonymous way, and participants are encouraged to revise their previous answers, reducing differences so that a convergence of opinions eventually develops. These methods can both effectively be used in medical and health service research and have been shown to give similar outcomes [25], but their use in combination has rarely been reported.

#### Study design

**Fig. 1:**
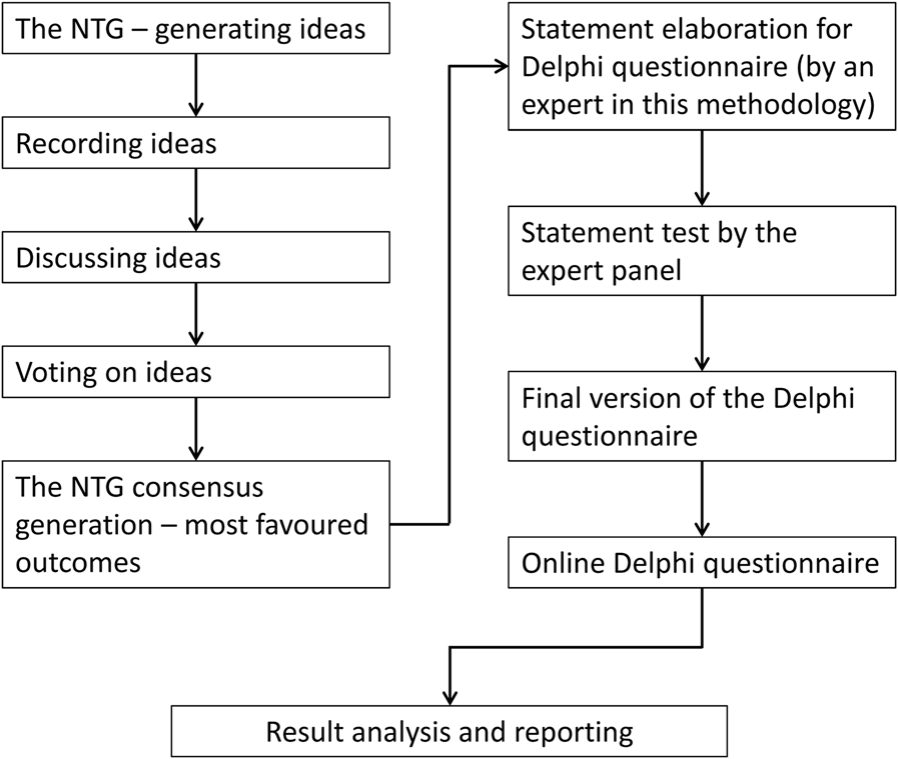
Overview of the study design. NTG, nominal group technique

The NGT was conducted following four steps: 1. Generating ideas: the problem “In your opinion what are the points worthy of study for the clinical-radiological definition of NSCLC?” was presented to the expert panel and the experts responded to it individually and independently by briefly outlining their ideas on paper; 2. Recording ideas: the ideas from the individual members of the panel were recorded, and overlapping ideas were deleted from the list; 3. Discussing ideas: each individual idea was discussed amongst the panel of experts to determine clarity and importance of the idea; 4. Voting on ideas: the discussed ideas were then individually prioritized by the experts. Each expert came up with five ideas they considered most important; the highest priority idea was rated as rank 5, and the least important as rank 1. The final responses from the experts were tallied to get the most favoured group outcomes on the problem. Thus, the NGT was used to gather information about the given question, and the issues that the expert panel considered relevant to answer this question were then used to develop the Delphi questionnaire that was submitted to the expert panel in a second phase. The overall study design is summarised in Fig. 1. Results were established based on the outcomes of the two methods.

## Results

### Nominal group technique

The NGT helped in identifying a broad area of important parameters for the evaluation of clinical and radiological features driving the therapeutic management of patients with NSCLC. From an original list of 20 parameters or “ideas”, the NGT led to the identification of six main areas: status of great vessels, status of lung parenchyma, non-morphological evaluation parameters of the tumour, organizational aspects, localisation of the tumour and tumour morphology. The ideas embodied in these broad areas were rated, establishing a limit cut-off consensus percentage of 66%. With these criteria, the expert panel nominated several points as relevant for the choice of antiangiogenic treatment in NSCLC. Central versus peripheral localization of the tumour, ratio of solid to cavitation component of tumours, presence of thromboembolism in great vessels and relationship to non-vascular adjacent thoracic structures were rated, in this order, as important by all the participants (100% consensus). Lung-vessels relationship was rated as important by five out of six participants, as well as the opportunity of a structured report, while presence of thromboembolism in peripheral vessels, use of standardized imaging techniques, general lung status regardless of the tumour presence and pleural effusion were rated, in this order, as important by four out of six participants.

### Delphi questionnaire

Based on the results obtained through the NGT, the Delphi questionnaire was set and answered by the expert panel to generate a consensus report. The results of the Delphi questionnaire are summarised in Table 1. The results of the questionnaire show that in order to prescribe an antiangiogenic therapy, information on the infiltration of the tumour into great vessels and mediastinum was considered most important by all the members of the expert panel. Complete consensus (100% agreement) was also reached regarding 5 mm thickness being inadequate to evaluate infiltration of vessels by computed tomography (CT) scan; the importance of having an awareness of the potential presence of a thrombus when considering antiangiogenic therapy; that pleural effusion is not always a contraindication to antiangiogenic therapy; that pleural effusion after pleurodesis is not a contraindication to antiangiogenic therapy; that, for the chest tumour site, a structured report is a useful tool when it contains information regarding cavitation, vascular infiltration, endobronchial growth and thromboembolism, and, for an extra-thoracic tumour site, information about thrombi, extra-thoracic sites of bleeding and the presence of aneurysms and brain metastases (Table 1).

**Table 1:** Delphi questionnaire consensus report

Statement		Respondents who agreed (%)
1	I feel that to implement an antiangiogenic therapy is important to know the infiltration of the tumour into adjacent structures such as:	
	i. Pleura	0.00
	ii. Chest wall	0.00
	iii. Bone	0.00
	iv. Bronchi	83.33
	v. Mediastinum	100.00
	vi. Oesophagus	66.67
	vii. Trachea	66.67
	viii. Carina	83.33
	ix. Large vessels	100.00
2	To evaluate vessels infiltration by CT scan in patients with NSCLC, I think it is sufficient to have the resolution given by a thickness of:	
	i. 5 mm	0.00
	ii. 3 mm	83.33
	iii. 1.5 mm	83.33
3	In the evaluation of treatment with antiangiogenic therapy I consider essential to know whether or not a thrombus is present	100
4	In the absence of infiltration of vessels, I think that tumour site (central or peripheral) is relevant for treatment	66.67
5	I think that cavitation is a contraindication to antiangiogenic therapy	66.67
6	I believe that the compression of a major vascular structure listed below by a secondary lymphadenopathy is a contraindication for antiangiogenic therapy	
	i. Vena cava	33.33
	ii. Aorta	16.67
	iii. Pulmonary arteries	33.33
	iv. Pulmonary veins	33.33
7	I think that proximity of the disease to a large vessel is not a contraindication to antiangiogenic therapy	66.67
8	I believe that the alteration of the lung parenchyma may be a risk factor for bleeding if it is:	
	i. Fibrosis	0.00
	ii. Bronchiectasis	50.00
	iii. Emphysema	0.00
	iv. Endobronchial tumour extension	83.33
	v. Pleural effusion	0.00
9	In evaluating the feasibility of antiangiogenic therapy I think it is essential to know the presence of deep venous thrombosis requiring antiplatelet therapy	83.33
10	I think that pleural effusion is always a contraindication to antiangiogenic therapy	0
11	I think that pleural effusion is a contraindication to antiangiogenic therapy only if it is haemorrhagic	50
12	I think pleural effusion is a contraindication to antiangiogenic therapy only after pleurodesis.	0
13	For the chest tumour site, I consider that, to be useful to clinical practice, a structured report should include at least:	
	i. Cavitation	100.00
	ii. Vascular infiltration	100.00
	iii. Fistulas	83.33
	iv. Endobronchial growth	100.00
	v. Lymphangitis	66.67
	vi. Margins	83.33
	vii. Thromboembolism	100.00
14	For the extrathoracic tumour site, I consider that, to be useful to clinical practice, a structured report should include at least:	
	i. Fistulas	66.67
	ii. Aneurysms	100.00
	iii. Diverticula	66.67
	iv. Extra-thoracic bleeding sites	100.00
	v. Brain metastases	100.00
	vi. Thrombi	100.00

Broad consensus (>80% agreement) was also reached on the following: the importance of information on infiltration into the bronchi and carina; that 1.5 mm or 3 mm thickness is sufficient for evaluating infiltration of vessels by CT scan; that compression of a vascular structure (particularly the aorta) is not a contraindication for antiangiogenic therapy; that endobronchial tumour extension may be a bleeding risk factor; that awareness of the presence of deep vein thrombosis requiring antiplatelet therapy is fundamental when considering antiangiogenic therapy; and that it is useful to have a structured report which contains information on fistulas and tumour margins (Table 1).

When considering whether the alteration of the lung parenchyma may be a risk factor for bleeding, the answers varied depending on whether the alteration was fibrosis, bronchiectasis, emphysema, endobronchial tumour extension, or pleural effusion (Table 1). There was no consensus regarding whether haemorrhagic pleural effusion is a contraindication to antiangiogenic therapy. The experts did agree that tumour localization in the absence of vessel infiltration, cavitation, and the use of antiplatelet therapy are relevant parameters to be assessed, but their presence should not necessarily exclude a patient from receiving antiangiogenic therapy.

## Discussion

Adding an antiangiogenic agent to standard chemotherapy provides substantial benefit in terms of response rate, progression-free survival and overall survival in advanced NSCLC; however, thorough evaluation of the patient as well as the tumour characteristics is needed to reduce the risk of serious complications such as PH [16]. The results of the present consensus addressed several key issues regarding the selection of patients with NSCLC for antiangiogenic therapy. Consensus output clearly stated that a detailed clinical and radiological analysis of the relationships between a tumour and its surrounding mediastinal structures is mandatory when considering an antiangiogenic therapeutic approach for the treatment of patients with NSCLC.

Tumour infiltration can be a risk factor for PH in patients with NSCLC undergoing antiangiogenic therapy [20]. The results of the survey suggested that evaluation of tumour infiltration in the mediastinum and major vessels, typically with a 1.5 mm CT scan resolution, is helpful in assessing the risk of PH when considering the antiangiogenic therapy. Tumour site in the absence of vessel infiltration and presence of a thrombus are important parameters for defining treatment selection. The contiguity of the tumour to a large vessel, however, is not a contraindication to antiangiogenic treatment, nor is the compression of a major vascular structure by a secondary lymphadenopathy. Moreover, pleural effusion is not always a contraindication to antiangiogenic therapy according to the experts involved in the study.

The expert panel also agreed that tumour cavitation is an important variable that should be examined before antiangiogenic therapy is used in NSCLC. It is essential to note that tumour cavitation is not in itself a contraindication to the use of antiangiogenic therapy, but is an important aspect to consider, since it is known to increase the risk of PH in patients with lung cancer [26]. Appropriate knowledge of any pre-existing tumour cavitation can be helpful in deciding the most appropriate course of treatment. The expert panel further suggested that it is essential to know of the presence of any deep vein thrombosis requiring antiplatelet therapy to prevent any further treatment-associated complications. In addition, among alterations of lung parenchyma (fibrosis, bronchiectasis, emphysema, endobronchial tumour extension, pleural effusion), the expert panel agreed that only an endobronchial tumour extension may be considered a risk factor for bleeding. However, neither the use of antiplatelet therapy nor tumour localisation should be considered contraindications to an antiangiogenic treatment approach.

One of the outcomes from the expert panel emphasised the importance and the need for a comprehensive and structured report. In fact, radiological reports lacking key information could lead to the exclusion of patients from antiangiogenic treatment. The expert panel agreed that a complete report should include information about cavitation, vascular infiltration, endobronchial growth, thromboembolism, margins, fistulas and lymphangitis, and a report for the extrathoracic tumour site should include at least information regarding aneurysms, brain metastases, thrombi, extra-thoracic bleeding sites, fistulas and diverticula. Such a treatment-oriented report, including a description of both tumour and general vascular status, is essential to guide the choice of treatment by the oncologist. Radiologists play an important role in diagnosis and assessment of treatment response in patients with NSCLC. Oncologists should share with radiologists parameters required for an appropriate risk-benefit evaluation of available treatments.

The main limitation of the present report is that only a small number of experts were included in the panel. Also, the proposed criteria for patient selection have not been validated in clinical practice. However, the criteria are based on the clinical knowledge and expertise of oncologists and radiologists and may be beneficial in reducing the risk of PH with the use of anti angiogenic therapy in patients with NSCLC.

## Conclusions

An expert panel comprising three oncologists and three radiologists examined important criteria/parameters when selecting patients with NSCLC for antiangiogenic therapy in order to ensure safety and in particular minimise the development of PH. Thorough clinical and radiological analysis of the relationships between tumour and vascular or anatomical structures (performed in close co-operation by oncologists and radiologists) was identified as an extremely important prerequisite. Before antiangiogenic treatment is used it is important to examine tumour cavitation, vascular infiltration, endobronchial growth and thromboembolism for chest tumour sites, and of the presence of aneurysms, extra-thoracic bleeding, brain metastases or thrombi for extra-thoracic sites. Overall, based on complete knowledge of relevant parameters, the co-operative efforts of radiologists and oncologists may provide a framework for the selection of patients and safe use of antiangiogenic agents for the treatment of NSCLC in a clinical setting.

## Abbreviations

CT: Computed tomography
MDCT: Multi-detector computed tomography
MR: Magnetic resonance
NGT: Nominal group technique
NSCLC: Non-small cell lung cancer
PET-CT: Positron emission tomography-computed tomography
PH: Pulmonary haemorrhage
VEGF: Vascular endothelial growth factor
